# Nanobody-Based Indirect Competitive ELISA for Sensitive Detection of 19-Nortestosterone in Animal Urine

**DOI:** 10.3390/biom11020167

**Published:** 2021-01-27

**Authors:** Yuan-yuan Yang, Yu Wang, Yi-feng Zhang, Feng Wang, Yi-fan Liang, Jin-yi Yang, Zhen-lin Xu, Yu-dong Shen, Hong Wang

**Affiliations:** 1 Guangdong Provincial Key Laboratory of Food Quality and Safety, National-Local Joint Engineering Research, Center for Processing and Safety Control of Livestock and Poultry Products, College of Food Science, South China Agricultural University, Guangzhou 510642, China; 835214940@stu.scau.edu.cn (Y.-y.Y.); 834670095@stu.scau.edu.cn (Y.-f.Z.); wangfeng_sp@163.com (F.W.); yfliang0605@163.com (Y.-f.L.); yjy361@163.com (J.-y.Y.); xzlin@scau.edu.cn (Z.-l.X.); 2 Guangzhou Institute of Food Inspection, Guangzhou 510080, China; xxwangyu@163.com

**Keywords:** 19-nortestosterone, nanobody, enzyme-linked immunosorbent assay, animal urine

## Abstract

Nanobody (Nb), a new type of biorecognition element generally from *Camelidae*, has the characteristics of small molecular weight, high stability, great solubility and high expression level in *E. coli*. In this study, with 19-nortestosterone (19-NT), an anabolic androgenic steroid as target drug, three specific Nbs against 19-NT were selected from camel immune library by phage display technology. The obtained Nbs showed excellent thermostability and organic solvent tolerance. The nanobody Nb2F7 with the best performance was used to develop a sensitive indirect competitive enzyme-linked immunosorbent assay (ic-ELISA) for 19-NT detection. Under optimized conditions, the standard curve of ic-ELISA was fitted with a half-maximal inhibitory concentration (IC_50_) of 1.03 ng/mL and a detection limit (LOD) of 0.10 ng/mL for 19-NT. Meanwhile, the developed assay had low cross- reactivity with analogs and the recoveries of 19-NT ranged from 82.61% to 99.24% in spiked samples. The correlation coefficient between ic-ELISA and the ultra-performance liquid chromatography/mass spectrometry (UPLC-MS/MS) method was 0.9975, which indicated that the nanobody-based ic-ELISA could be a useful tool for a rapid analysis of 19-NT in animal urine samples.

## 1. Introduction

Nandrolone phenylpropionate (NPP), one of commonly used steroid hormones in foodborne animal, has strong effect of protein assimilation. It has been extensively used in livestock breeding for accelerating muscle growth with supernormal pattern and fat storage reduction for improving the economic benefit of feed [1]. However, the abuse of NPP would cause various degrees of residue in animal tissues. The secondary metabolite 19-NT is main residual marker which has strong stability and long half-life period in animals [2]. 19-NT was confirmed to have a series of adverse effects on human health, such as adrenal atrophy, endocrine disorders, the damages in liver function, cardiovascular system, reproductive organ and so on [3,4,5,6]. Since 1986, many countries and regions including European Union and China, have gradually banned the addition of synthetic protein hormones in food-borne animals [7]. However, the metabolic residues of anabolic steroid were frequently detected in animal-derived food. Therefore, it is urgent to develop an economic, sensitive and efficient detection method for the metabolite of anabolic androgenic steroid.

Enzyme-linked immunosorbent assay (ELISA) has been an ideal based on antigen-antibody specific recognition. It has been widely used in the detection of small molecule contaminants in foodstuffs because of its advantages of convenient operation, excellent sensitivity and specificity [8,9]. Until now, there have only been a few immunoassay reports on 19-NT and reported studies were generally based on polyclonal antibodies (pAbs) or monoclonal antibodies (mAbs) [10,11,12]. With the development of engineering antibodies, nanobodies (Nbs), the variable domains derived from heavy chain antibodies generally present in camelids, are getting more and more attention for its advantages over traditional pAbs or mAbs. As a smallest antibody fragment with complete antigen binding function, Nbs have excellent physicochemical stability that allow them to work at higher temperatures or organic solvent and is easy to be genetically manipulated with high yield [13]. Based on these advantages, Nbs have been applied in various immunoassays for small molecule analytes [14], including *Alternaria* mycotoxin tenuazonic acid [15], aflatoxin [16], parathion [17], carbofuran [18] and microcystin-LR [19].

In this study, three highly specific Nbs against 19-NT were isolated from an immune nanobody phage library. To explore their potential in immunoassay, we evaluated the physical-chemical properties of Nbs and the best one nanobody Nb2F7 was used to develop an ic-ELISA for 19-NT in spiked animal urine samples. Finally, the immunoassay results were applied in actual samples detection and validated by the ultra-performance liquid chromatography/mass spectrometry (UPLC-MS/MS).

## 2. Materials and Methods

### 2.1. Reagents and Materials

**Figure 1:**
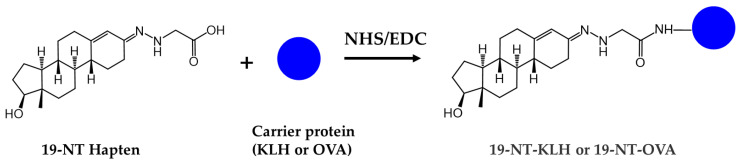
Structures of the conjugates of hapten 19-NT with carrier protein. 19-NT hapten, EDC (1-(3-Dimethylaminopropyl)-3-ethylcarbodiimide hydrochloride) and NHS (N-Hydroxy succinimide) were dissolved in 0.5 mL DMF(N,N-Dimethylformamide) (hapten: EDC: HNS = 1:1.5:1.5, molar ratio) and stirred in darkness for 4 h. Then OVA or KLH was dissolve in CB (Carbonate Buffer, pH 9.6) to 10 mg/mL final concentration and added to hapten-EDC/NHS dropwise (hapten: OVA/KLH = 30:1, molar ratio). The mixture was stirred in darkness overnight and then dialyzed in PBS to remove the unbinding hapten [20].

The 19-nortestosterone, estradiol (E2), trenbolone (TRE), methyltestosterone (MT) and testosterone propionate (TP) were purchased from Dr. Ehrenstorfer GmbH (Augsburg, Germany). testosterone (TES) and nandrolone phenylpropionate (NPP) were purchased from MACKLIN (Shanghai, China). The keyhole limpet hemocyanin (KLH), bovine serum albumin (BSA), ovalbumin (OVA), Freund’s complete adjuvants and Freund’s incomplete adjuvants were purchased from Sigma (St. Louis, MO, USA). The conjugates 19-NT-KLH and 19-NT-OVA were prepared in our laboratory (Figure 1). The TRIzol reagent was from Thermo Fisher Scientific (Shanghai, China). The first strand cDNA synthesis kit was purchased from TaKaRa (Dalian, China). The gel extraction and PCR purification kit were obtained from QIAGEN (Dusseldorf, Germany). The molecular biology reagents, including helper phage M13K07, restriction enzymes, T4 DNA ligase and others, were purchased from New England Biolabs (Ipswich, MA, USA). The rabbit anti-camelid VHH antibody-HRP (anti-VHH-HRP) was purchased from GenScript (Nanjing, China).

### 2.2. Construction of Phage Displayed Nanobody Library of Nanobody Library.

A three-year-old *Bactrian* camel was immunized subcutaneously with the mixture of 500 μg 19-NT-KLH and Freund’s adjuvant biweekly. 100 mL of blood was collected for isolation of PBMC (Peripheral Blood Mononuclear Cell) and extraction of total RNA to perform reverse transcription. The VHH genes were amplified by two-step nested PCR [21] using the primers CALL001 (5′-GTCCTGGCTGCTCTTCTACAAGG-3′) and CALL002 (5ʹ-GGTACGTGCTGTTGAACTGTTCC-3′) for first step amplification of VH and VHH genes and the primers *Sfi*-Fr1 (5′-ACTGGCCCAGGCGGCCGAGGTGCAGCTGSWGSAKTCKG-3′) and *Sfi*-Fr4 (5′-ACTGGCCGGCCTGGCCTGAGGAGACGGTGACCWGGGTC-3′) for amplifying the VHH regions. The pComb3XSS phagemid vector and VHH genes were digested with *Sfi*I restriction enzyme and ligated by T4 DNA ligase. The ligation products were purified and transformed into *E. coli* TG1 competent cells. After cultured and infected with helper phage M13KO7, the phage library was obtained for biopanning.

### 2.3. Construction and Screening of Phage Display Library

The library was subjected to four rounds of panning on 96-well microtiter plates. A series of decreasing concentrations of 19-NT-OVA (12, 3, 0.75 and 0.1875 μg/mL, 100 µL/well) were incubated overnight in different wells of plates at 37 °C, followed by switched blocking buffers (1% fish gelatin or 2% skimmed milk in PBS) for avoiding the non-specific binding. Additional wells coated with different carrier proteins (1 μg/mL KLH, BSA and OVA) was designed and conducted to eliminate the phage non-specific binding. After washing twice with PBST (0.5% Tween 20), the wells were blocked with 300 µL/well blocking buffer and incubated at 37 °C for 3 h. During the procedures of panning, 100 µL/well phage library was added into the carried protein coated-wells and incubated at 37 °C for 1 h. The nonbound phages were transferred into 19-NT-OVA-coated wells and incubated at 37 °C for another 1 h. The plate wells were washed 15 times with PBST and PBS, the competitive elution was performed with different concentrations of 19-NT (100 µL/well) (1.2, 0.3 and 0.15 µg/mL), respectively. 10 µL eluted phages were used to test the titer and the residues were amplified for the next round of panning. After four rounds of panning, the clones were randomly selected and cultivated individually overnight at 37 °C for nanobody expression. The supernatants were analyzed by ELISA. The plasmids of positive clones were extracted and sequenced for further research.

### 2.4. Preparation and Identification of Anti-19-NT Nanobody

The plasmids of the 19-NT specific clones were extracted and chemically transformed into *E. coil* BL21(DE3) competent cells. After sequencing and identification, a single clone was picked up and cultured in LB medium (100 µg/mL ampicillin) overnight. The precultured cells were inoculated in 2×YT liquid medium (100 µg/mL ampicillin) at 37 °C with 250 rpm shaking to reach the absorbance value of 0.6–0.8 at the wavelength of 600 nm. Then the cells were induced by 1 mM IPTG overnight at 37 °C and 220 rpm. The next day, cell pellets were collected by 12,000 rpm centrifugation for 10 min and the His-tagged Nb was extracted by sucrose osmotic pressure method and purified by Ni-NTA affinity chromatography [22]. The purified Nb was identified by 12% SDS-PAGE and the concentration was measured using a NanoDrop 2000C system.

### 2.5. Stability Analysis of Anti-19-NT Nanobody

In order to evaluate the stability of the Nb2F7, Nb2B8 and Nb3D10, the purified Nbs (1 mg/mL) were incubated at 25 °C, 35 °C,50 °C, 60 °C, 70 °C, 80 °C and 90 °C for 5 min. Each of Nb samples were re-equilibrated to room temperature (RT), followed by assessment for their binding activity. And a series concentration (0%, 10%, 20%, 40%, 60%, 80%, 100%) of methanol (MeOH) and acetonitrile were used as the dilution reagents (*v*/*v*) for assessment of organic solvents tolerance. The binding activity of Nbs were determined by ELISA under varying temperatures or organic solvents [23].

### 2.6. Development and Optimization of ic-ELISA Based on Nanobody

The ic-ELISA was developed with a competitive strategy. A 96-well microplate was coated with 100 μL/well of 1 μg/mL 19-NT-OVA in coating buffer at 37 °C overnight, followed by twice washing with PBST and blocking with 2% skimmed milk in PBS (150 μL/well) at 37 °C for 3 h. 50 μL of sample solutions or standard solutions standard 19-NT solution diluted in PBS (Phosphate Buffered Saline) and 50 μL of 156.25 ng/mL Nb was added and mixed gently. After a 40 min-incubation at 37 °C, the plate was washed with PBST for five times and added with 100 μL/well anti-VHH-HRP (1:5000) in PBST (0.05% Tween 20) for 40 min at 37 °C. 100 μL of TMB peroxidase substrate solution was added in the wells after five times of washing and the reaction was stopped after 10 min incubation by adding 50 μL of 10% H_2_SO_4_. The standard curve was fitted with a four-parameter fitting module of Origin 8.5 (Origin Lab Corp, Northampton, MA, USA). The value of “B/B0” was used to characterize the binding ability of nanobodies to the coating antigen on the microplate, where B and B0 represented the absorbance values in the presence or absence of 19-NT, respectively. The half maximal inhibitory concentration (IC_50_) and limit of detection (LOD) were defined as the concentration of 19-NT that produced 50% and 10% inhibition rate. The percentage of cross reactivity was calculated as follows: CR (%) = IC_50_ (19-NT, ng/mL)/IC_50_ (analogues, ng/mL) × 100.

Reaction conditions have a great influence on the sensitivity of immunoassays. Therefore, the parameters were investigated including ionic concentration in dilution buffers, concentration of anti-VHH-HRP and reaction times in different incubation steps. The Amax (the maximum relative absorbance value), IC_50_ and Amax/IC_50_ were used to evaluate the factors as previously described [15].

### 2.7. Real Sample Analysis by ic-ELISA and UPLC-MS/MS

The urine samples from bovine and swine were collected from Wens Food Group Co. LTD (Guangzhou, China) and Dinghu dairy farm (Zhaoqin, China). 10 mL urine sample was centrifuged at 4 °C with 3500 rpm for 15 min and the supernatant layer was filtered through a 0.22 μm filter. Afterwards, bovine urine samples were spiked with three different concentrations of 19-NT (10, 20 and 40 ng/mL) and pig urine samples were proceeded with 20 ng/mL, 40 ng/mL and 80 ng/mL of 19-NT. The urine was diluted with PBS for sample analysis by ic-ELISA.

For UPLC-MS/MS validation, it was different from the previous operation. Briefly, 5 g of a urine sample (accurate to 0.01 mg) was weighed in a 50 mL centrifuge tube and mixed with a 19-NT standard solution. After the addition of 10 mL acetonitrile, the centrifuge tube was vortexed for 1 min. An extract salt pack (containing 4 g magnesium sulfate, 1 g sodium citrate,1 g sodium chloride and 0.5 g disodium hydrogen citrate) was added, shaken for 1 min and centrifuged at 6000 rpm for 3 min. 5 mL of the upper organic layer was transferred into a 15 mL centrifuge tube containing 50 mg PSA, 150 mg C18 and 900 mg anhydrous sodium sulfate, vortexed for 3min and centrifuged at 6000 rpm for 3 min. The 1 mL supernatant was directly diluted five times with distilled water and passed through a 0.22 μm filter to obtain the sample. The conditions were used as follows: mobile phase A consisted of 0.1% formic acid and 5 mmol/L ammonium formate in methanol and mobile phase B consisted of 0.1% formic acid and 5 mmol/L ammonium formate in water. The gradient elution was 0–1 min, 80% B; 1–4 min, 80% B-30% B; 4–11 min, 30% B-0; 11–12 min, 0 B; 12–12.5 min, 80% B; and 12.5–15 min, 80% B. The flow rate of the mobile phase was 0.3 mL/min and an aliquot of 5 μL of each sample was injected into the UPLC system. The mass spectra were obtained with AB TRIPLE QUAD 4500 mass spectrometer using the electrospray ionization technique and the 19-NT samples were analyzed in the positive ionization mode.

## 3. Results and Discussion

### 3.1. Screening and Identification of Nanobody

**Figure 2:**
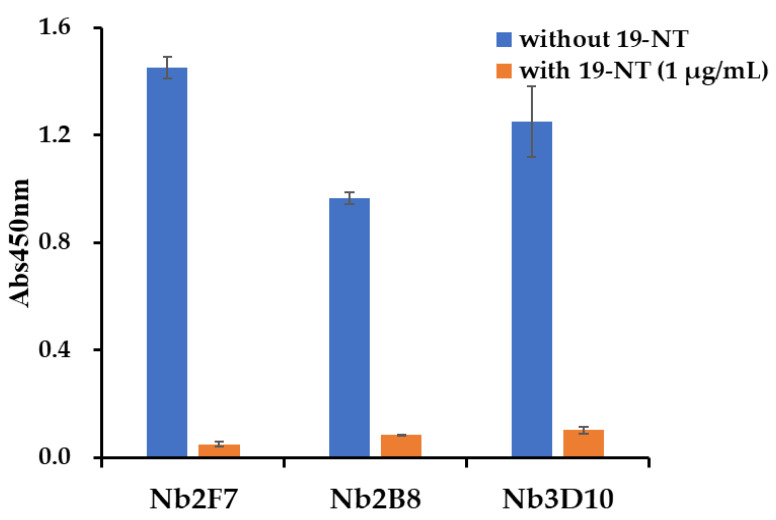
Positive clones identified by indirect competitive enzyme-linked immunosorbent assay (ic-ELISA) analysis of the expression supernatant.

**Figure 3:**
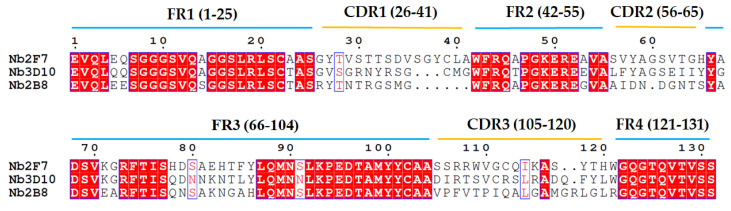
Sequence alignment of three positive clones.

**Figure 4:**
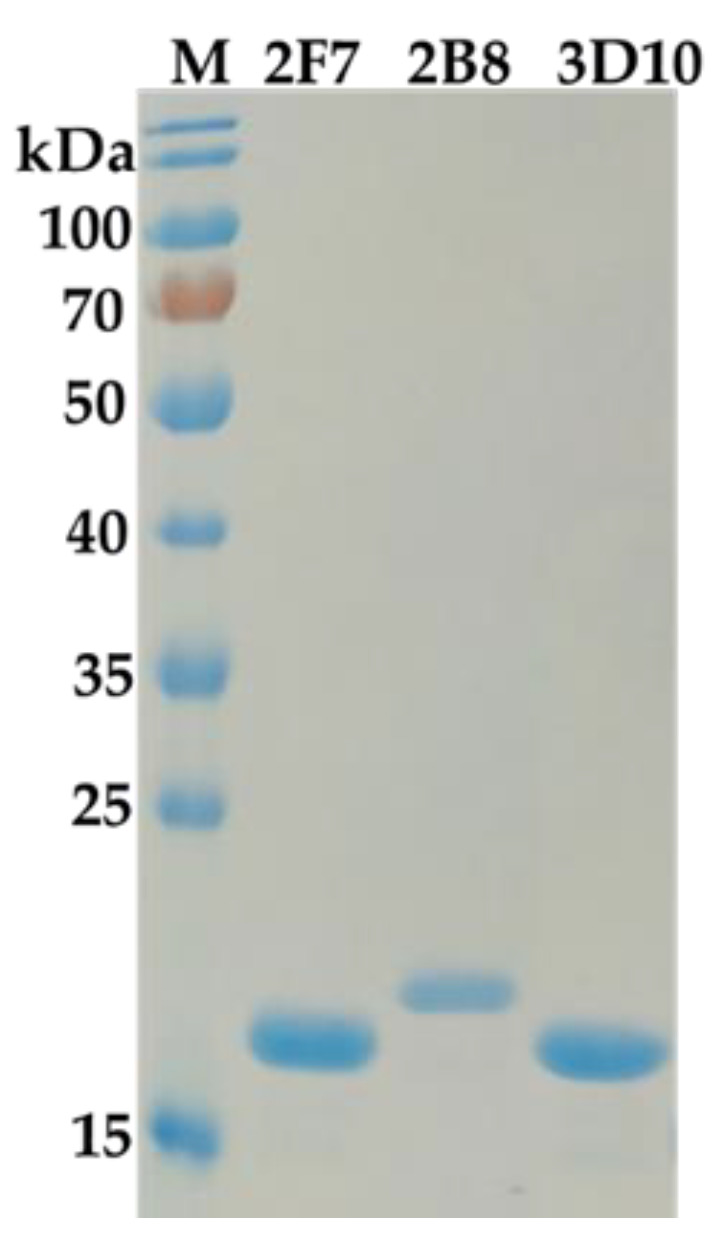
Characterization of nanobodies by SDS-PAGE.

The capacity of Nb library was 5.78 × 10^7^ cfu/mL. The titers of phage display Nb library was up to 1.55 × 10^13^ pfu/mL after rescuing by helper phage M13K07. Four rounds of biopanning were conducted with the immobilization of 19-NT-OVA and enrichment of target fragments started from the second round biopanning. After four rounds of biopanning, forty valid positive clones were finally obtained. According to the sequence difference in CDR regions, all these positive clones could be divided into three subfamilies and the inhibition rates for 19-NT were within the ranged from 76.02% to 96.63% under 1 µg/mL of 19-NT. Three excellent nanobodies named Nb2F7, Nb2B8 and Nb3D10 were selected from the three families with inhibition rates of 96.63%, 91.30% and 91.79%, respectively (Figure 2). Although all these nanobodies had high similarity in the framework regions (FRs), however the complementarity determining regions (CDRs) varied widely (Figure 3). Then Nb2F7, Nb2B8 and Nb3D10 were expressed in *E. coli* BL21(DE3) with the yields were of 10 mg/L, 7.3 mg/L and 5.7 mg/L, respectively. Results in SDS-PAGE (Figure 4) showed that the size of these Nbs ranged about 18 kDa.

### 3.2. Stability Analysis of Anti-19-NT Nanobody

**Figure 5:**
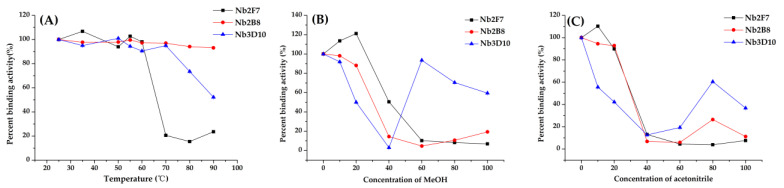
Thermostability and organic solvents tolerance of Nbs by ELISA based on the antigen 19-NT-OVA and anti-VHH-HRP antibody. (**A**) anti-19-NT Nbs (1 mg/mL) was incubated at 25, 35, 50, 55, 60, 70, 80 and 90 °C for 5 min. A series concentration (10%, 20%, 40%, 60%, 80% and 100%) of (**B**) MeOH, (**C**) acetonitrile.

Due to their unique structure, Nbs are robust under harsh conditions and can resist chemical and thermal denaturation. In this work, evaluation of thermal stability and organic solvent tolerance was performed to characterize the Nb2F7, Nb2B8 and Nb3D10. The results of thermostability study (Figure 5A) showed that three Nbs could maintain nearly 100% activities after being incubated at 60 °C for 5 min. In addition, the remained binding activity of Nb2B8 and Nb3D10 was over 50% after incubated at 90 °C for 5 min. Disulfide bonds are known as a major factor influencing the heat resistance of proteins [24,25]. This phenomenon may be related to intramolecular disulfide bonds in the complementary determination region of Nbs [26,27]. Moreover, the Nb2F7 incubated at a temperature higher than 60 °C for 5 min, the antigen-antibody binding activity remained only about 20%. It may induce the destruction or unfolding of the VHH structure after the thermal induction process [28,29].

**Figure 6:**
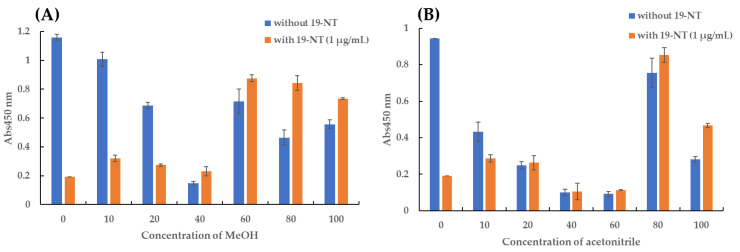
Binding activity of Nb3D10 in organic solvent by ELISA. A series concentration (0%, 10%, 20%, 40%, 60%, 80% and 100%) of (**A**) MeOH, (**B**) acetonitrile.

Furthermore, the organic solvent tolerance is also an index to evaluate the performance of Nb. The three Nbs exhibited superior tolerance under high concentrations of methanol and acetonitrile. The binding activity of Nb2F7 and Nb2B8 was maintained above 88% under the treatment of 20% MeOH or acetonitrile in PBS and the binding activity of Nb3D10 was about 50% (Figure 5B,C). Moreover, we also found a strange phenomenon that the binding activity of all Nbs decreased with the increase of organic solvent concentration, while Nb3D10 showed an upward trend when the organic solvent was higher than 40%. To further study this phenomenon, 50 μL Nb3D10 was combined with 50 μL PBS-organic solvents presented or absented of 19-NT and performed ELISA. The results showed that with the increase of the concentration of the organic solvent, the Nb3D10 cannot be completed by 19-NT anymore during ic-ELISA analysis but a high A_450nm_ value appeared (Figure 6). The reason might be that high concentration organic solvents lead to the structure change of the coating antigen-antibody complex so that Nb3D10 lost the ability to recognize free 19-NT but the complex could still be combined by the anti-VHH-HRP [30,31]. However, the mechanisms are not very clear yet. Overall, Nbs exhibited good stability even though under the effect of extreme temperature and high concentrations of organic solvents.

### 3.3. Establishment of ic-ELISA Method for 19-NT Based on Nanobody

**Figure 7:**
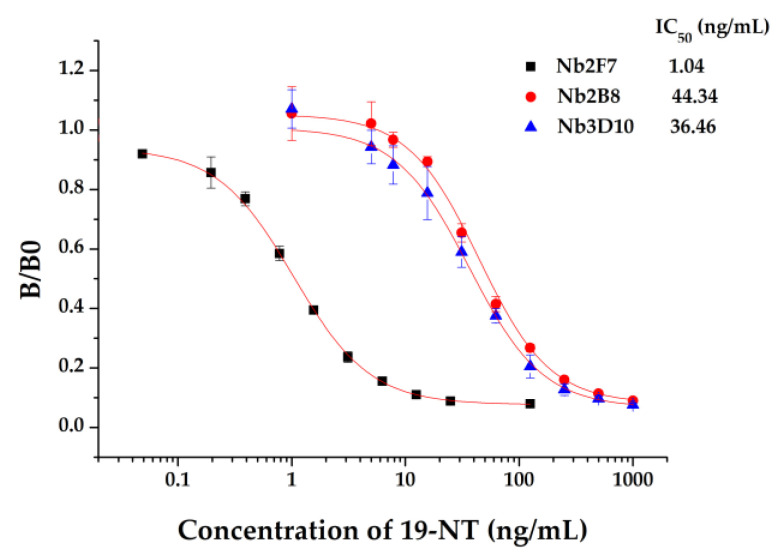
Standard curves of ic-ELISA based on Nb2F7, Nb2B8 and Nb3D10.

The sensitivity of Nb2F7, Nb2B8 and Nb3D10 were evaluated by establishing standard curves (Figure 7). The three Nbs exhibited a different property, with IC_50_ values of 1.04 ng/mL, 44.34 ng/mL and 36.46 ng/mL. The difference of sensitivity of the three nanobodies is due to their different sequences (Figure 3) and especially the CDRs play an important role [32,33]. The specificity of the proposed assay was evaluated by using six analogs as competitors. As show in Table 1, all Nbs exhibited a similar CRs for TRE, TES and E2 and neglectable CRs (<0.1%) for MT, TP and NPP. This was probably explained by the obvious effect of benzene ring and hydroxyl group in affinity of the steroids. Similarly, the difference in shapes, volumes and electrostatic potentials of compounds structure might also be the influence factors [34]. In terms of the sensitivity and specificity for 19-NT recognition, the Nb2F7 was selected for further study.

### 3.4. Optimization of ic-ELISA

**Figure 8:**
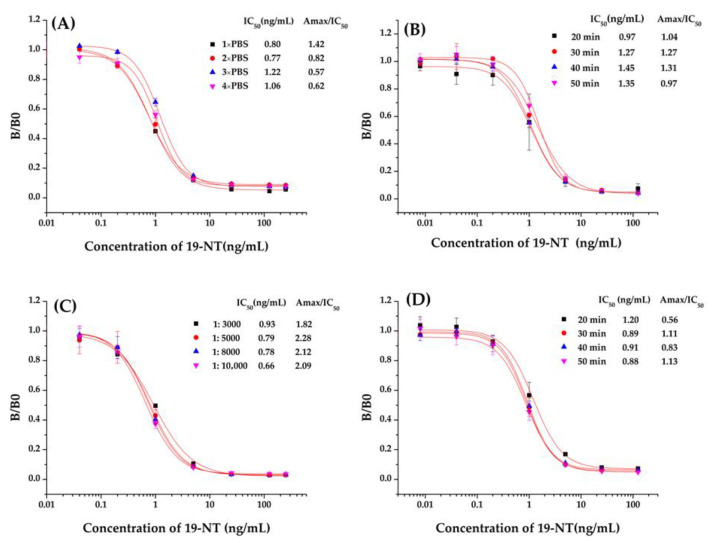
Parameters optimization of ic-ELISA based on Nb2F7. (**A**) Concentrations of PBS, (**B**) Incubating time of anti-19-NT Nb, (**C**) Concentrations of anti-VHH-HRP, (**D**) Incubating time of anti-VHH-HRP.

The developed ic-ELISA based on Nb2F7 was optimized with the proper experimental conditions. Through a checkerboard test, the optimal concentrations of coating antigen and Nb2F7 were firstly confirmed, then under the condition of 250 ng/mL of coating antigen and 156.25 ng/mL of Nb2F7, other reaction condition including ionic concentration of PBS, reaction times in different incubation steps and concentration of anti-VHH-HRP were optimized. The results of single factor experiment of different experimental conditions are shown in Figure 6. Both the IC_50_ value and Amax /IC_50_ slightly fluctuated as the ionic strength changes and 1× PBS of ionic strength was selected for further research (Figure 8A). Next-step assays were performed with various Nb incubation time (Figure 8B). The IC_50_ value increased from 0.97 to 1.45 ng/mL as the incubation time increased from 20 min to 40 min. So, the incubation time of 20 min was selected for the optimal factor. We also studied the influence of the anti-VHH-HRP concentrations and incubation time (Figure 8C,D). Compared with IC_50_ value and Amax /IC_50_, the best dilution ratio and incubation time of anti-VHH-HRP were finally selected as 1:10,000 and 30 min, respectively.

**Figure 9:**
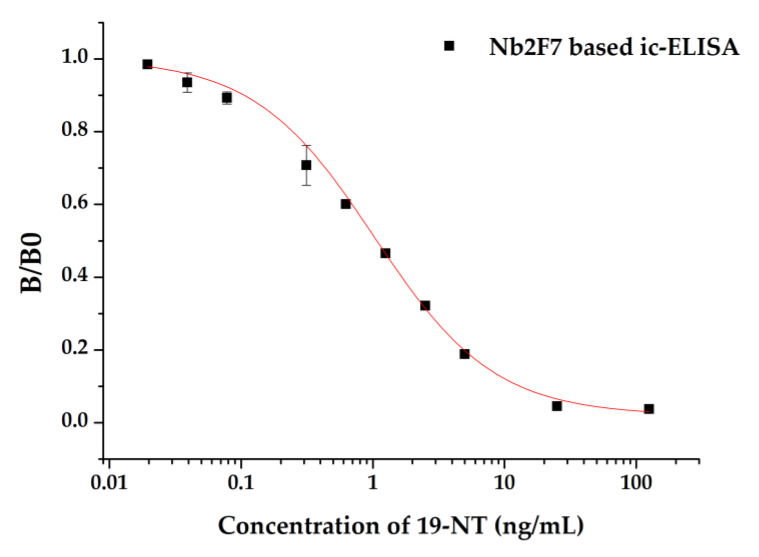
Standard competitive inhibition curve for 19-NT analysis.

Under the optimal conditions, a calibration curve was established with the IC_50_ of 1.03 ng/mL and LOD of 0.10 ng/mL for 19-NT (Figure 9). Meanwhile, the ic-ELISA described in this study is more sensitive than other immune methods [12,34,35,36] show in Table 2. Also, a monoclonal antibody-based ELISA for 19-NT was constructed with the IC_50_ value of 0.55 ng/mL [10]. But compared with the production of monoclonal antibodies and polyclonal antibodies, the specific nanobodies obtained by the phage display technology present high sensitivity and great prospects in immunoassay for detection of 19-NT [37].

### 3.5. Sample Analysis by ic-ELISA and UPLC-MS/MS

**Figure 10:**
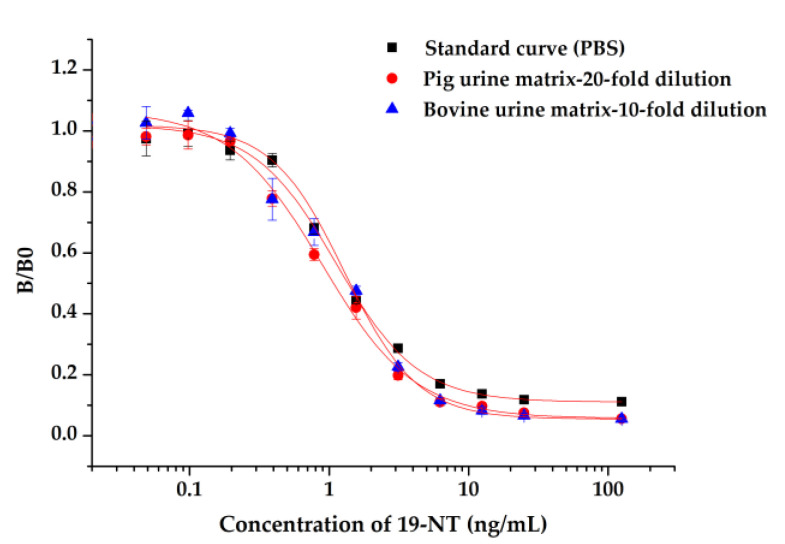
Sample matrix extracts of ic-ELISA.

**Figure 11:**
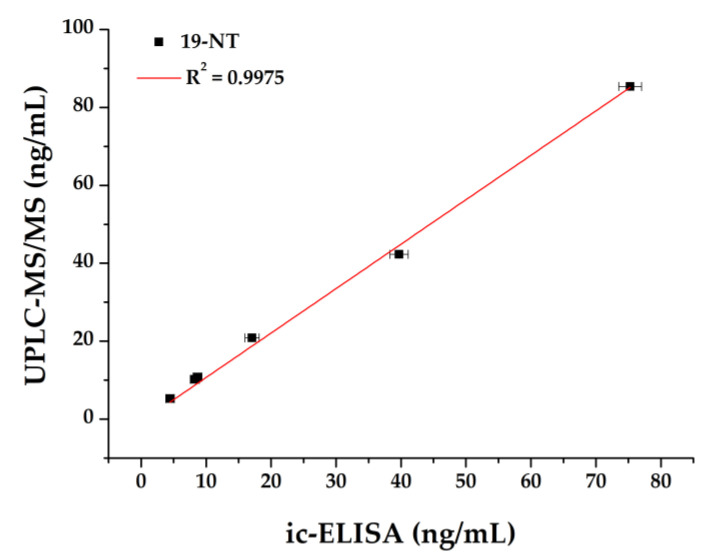
Correlations of analysis of samples spiked with 19-NT between ic-ELISA and UPLC–MS/MS.

Nb2F7-based ic-ELISA was employed to analyze pig and bovine urine samples spiked with 19-NT at three concentrations. To effectively eliminate interference of sample matrix, several standard curves using series of dilutions of pig and bovine urine as diluent were established and compared with the original curve with PBS. It was indicated that the matrix effects were largely minimized with 10-fold dilution of bovine urine and 20-fold dilution of pig urine (Figure 10). The sample extractions were analyzed by ic-ELISA under optimal reaction conditions. Results in Table 3 indicated that the average recoveries ranged from 82.61% to 99.24% with the coefficient of variation (CV) lower than 15% in spiked samples, which could meet the detection requirements of actual samples. To evaluate the accuracy of ic-ELISA method, the contents of 19-NT in samples were also validated with UPLC-MS/MS. The correlation coefficient of ic-ELISA and UPLC-MS/MS was 0.9975 (Figure 11). As the results, the proposed nanobody-based ic-ELISA for 19-NT was demonstrated to be a reliable technology for real sample analysis.

## 4. Conclusions

In this study, three specific Nbs against 19-NT were successfully selected from the camel immune nanobody library and their characteristics were identified. Then, a series performance analysis based on the selected Nbs were carried out. The results showed that the Nb2F7 had better thermostability, higher tolerance to organic solvents and especially good sensitivity and specificity for 19-NT. An ic-ELISA method for the detection of 19-NT in animal urine was further developed using Nb2F7, which had high sensitivity for 19-NT detection and acceptable recoveries in urine samples. The proposed method was verified by UPLC-MS/MS with a good correlation coefficient. Therefore, the nanobody-based immunoassay can be regarded as an ideal primary screening method for the detection of 19-NT residues in animal urine. At the same time, this simple, inexpensive and sensitive ic-ELISA program is expected to provide a technical support for the monitoring of such steroid hormones.

## Figures and Tables

**Table 1:** Cross-reactivity of Nbs with 19-NT structural analogues.

Analytes	Structure	Nb2F7 (CR)	Nb2B8 (CR)	Nb3D10 (CR)
19-NT	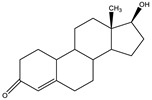	100%	100%	100%
TER	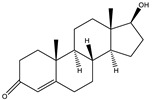	5.67%	45.32%	8.49%
E2	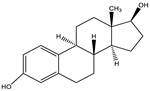	0.19%	38.48%	<0.1%
TRE	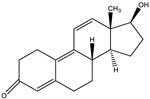	11.24%	22.47%	13.52%
MT	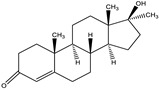	<0.1%	<0.1%	<0.1%
TP	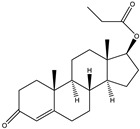	<0.1%	<0.1%	<0.1%
NPP	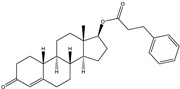	<0.1%	<0.1%	<0.1%

**Table 2:** Sensitivity of Reported Anti-19-NT Reagents.

Antibody	Detection Method	IC_50_ (ng/mL)	LOD (ng/mL)	Reference
pAb	ic-ELISA	27	1.9	[28]
pAb	ELISA	1.97	0.013	[29]
pAb	ELISA	6.41	0.09	[30]
pAb	strip	-	5	[31]
mAb	ic-ELISA	0.55	0.002	[10]
Nb	ic-ELISA	1.02	0.10	this work

**Table 3:** Recovery Analysis of 19-NT in the Spiked Samples by ic-ELISA.

Sample	Spiked Level (ng/mL)	Found ± SD (ng/mL)	Recovery (%)	CV (%)
bovine urine	5	4.64 ± 0.66	92.82	14.25
	10	8.67 ± 0.68	86.87	7.82
	40	39.69 ± 1.39	99.24	3.50
pig urine	10	8.26 ± 0.69	82.61	8.39
	20	17.07 ± 0.50	86.87	6.43
	80	75.26 ± 1.16	94.08	2.32

## Data Availability

Not applicable.
